# A three-dimensional lattice-free agent-based model of intracellular ice formation and propagation and intercellular mechanics in liver tissues

**DOI:** 10.1098/rsos.231337

**Published:** 2024-07-17

**Authors:** Fatemeh Amiri, James D. Benson

**Affiliations:** Department of Biology, University of Saskatchewan, Saskatoon, Saskatchewan, Canada

**Keywords:** cryobiology, ice formation, agent-based model, Monte Carlo, ice propagation, tissue biomechanics

## Abstract

A successful cryopreservation of tissues and organs is crucial for medical procedures and drug development acceleration. However, there are only a few instances of successful tissue cryopreservation. One of the main obstacles to successful cryopreservation is intracellular ice damage. Understanding how ice spreads can accelerate protocol development and enable model-based decision-making. Previous models of intracellular ice formation in individual cells have been extended to one-cell-wide arrays to establish the theory of intercellular ice propagation in tissues. The current lattice-based ice propagation models do not account for intercellular forces resulting from cell solidification, which could lead to mechanical disruption of tissue structures during freezing. Moreover, these models have not been expanded to include more realistic tissue architectures. In this article, we discuss the development and validation of a stochastic model for the formation and propagation of ice in small tissues using lattice-free agent-based model. We have improved the existing model by incorporating the mechanical effects of water crystallization within cells. Using information from previous research, we have also created a new model that accounts for ice growth in tissue slabs, spheroids and hepatocyte discs. Our model demonstrates that individual cell freezing can have mechanical consequences and is consistent with earlier findings.

## 1. Introduction

The application of cryobiology in science and medicine has been a great success and includes preserving blood, gametes and cell therapeutics, but also cartilage, heart valves, corneas and other small tissues [1–8]. Unfortunately, most tissues and all organs cannot currently be successfully cryopreserved [9]. In fact, there are only a few preliminary and limited reports of successful organ preservation in the absence of ice for a period of less than a week [10]. In short, there is a worldwide need for improvement in the recovery of tissues and organs after cryopreservation.

Physical injury to biological cells during freezing can be caused by ice crystalization, inter- and intracellular forces that arise from the expansion of water into ice, or the extreme dehydration and associated high salt concentrations due to the formation of extracellular ice that provides a driving force for both dehydration of the cell and for the nucleation and growth of damaging intracellular ice [11]. Theoretical methods have been developed to improve the design of cryopreservation protocols and minimize the damage created by these factors. For example, the water transport model that was developed by Mazur [12] and later modified by Levin [13] captures the dynamics of the water volume of the cells during cooling at rates where the extracellular medium is not supercooled. Mazur proposed that this model was useful because damaging intracellular ice formation (IIF) could be avoided by cooling cells slowly enough so that the osmotic dehydration allows the intracellular milieux to remain in chemical potential equilibrium with the external environment. In fact, the definition of ‘slowly enough’ could be tested theoretically using the model, and this was borne out in experiments that would go on to support one side of Mazur’s ‘two factor hypothesis’ [14]. Mazur concluded that the alignment with experiments suggested that cells with less than 2°C of theoretically predicted supercooling were not likely to have IIF [14].

This work to predict IIF was refined significantly with a statistical approach for predicting the likelihood of intracellular ice, introduced by Pitt [15], that had some success in approximating the probability of IIF in different cell types. However, Pitt’s model does not provide information about the mechanisms of ice nucleation in the supercooled cytoplasm. Following Pitt, Toner *et al.* developed a physico-chemical theory of heterogeneous nucleation in cells based on a thermodynamic model of ice nucleation, coupled with Mazur’s model of cell dehydration during freezing [16]. Finally, Karlsson *et al.* [17,18] further refined Toner’s model by adding diffusion-limited growth that depended on intracellular viscosity, an important consideration in the multimolar cryoprotectant concentrations that exist in sub-zero temperatures during cryopreservation.

Although these models were successfully implemented in individual cells [19,20], cryomicroscopic observations indicated that the probability of IIF in cells in cell monolayers, colonies and tissues is higher than the probability of IIF in suspended cells of the same type [21]. These findings suggest that cell–cell interaction plays a major role in intercellular ice nucleation. To address this with a mathematical model, Irimia and Karlsson extended the isolated cell IIF models to account for the propagation of IIF from one cell to a neighbouring cell using a Markov process for a simple two cell construct, and successfully validated their theory with experiment [22]. Ostensibly, this was the first cell-based model of ice propagation in tissues, and Irimia and Karlsson then extended this two-cell model and experiments to linear four-cell arrays [23]. In these cell-based models, the Markov chain and the resultant ordinary differential equation (ODE) were solved for the probabilities of each IIF outcome of the tissue. Because the state space of the generator matrix in a Markov chain model grows exponentially with the number of cells [24], a numerical Monte Carlo approach was also implemented and tested in linear arrays of up to 100 cells. Finally, work extending to a two-dimensional array was presented in an otherwise unpublished master’s thesis [25] and short conference abstracts [26], and an exploration of ice propagation in a somewhat unrealistic and spherically symmetric tissue [27,28].

These small-scale lattice-based tissue ice propagation models have not been extended to more realistic tissue structures, and do not account for intercellular forces that arise from the expansion of water into ice that may cause mechanical disruption during freezing. Moreover, the number of intercellular connections are three to four times higher in a three-dimensional tissue, and as such the intercellular ice propagation rates and the importance of the types of connections governed by cell type and structures will play an important and unexplored role in the ice dynamics in these tissues. Additionally, as some cell types and structures may tolerate intracellular ice, while others do not, the location of each individual cell type and the likelihood that intracellular ice will cause damage should allow modelling of whether tissues will lose viability during any specific cooling protocol. Finally, the complex interaction of heat and mass transfer and the resultant IIF in large tissues plays a critical role during freezing, and is coupled with cell and tissue level osmotic response or temperature change, that, for example, is delayed at the centre of a tissue compared to that at the tissue boundary [13,29].

While Karlsson, Irimia and Sumpter stopped at one- and two-dimensional structures for their cell-based models, there have been a number of other attempts to capture the three-dimensional propagation of ice in tissues and organs. These have used continuum models such as the Johnsson–Avrami–Mehl–Kolmagorov model that generalizes the classic Stefan problem of the propagation of a phase transition through a homogeneous material [30]. In the case of cryobiology, these models have been mostly applied to understanding the extents of ice balls formed during ablative cryosurgery [31,32]. They were also applied and compared with the cell-based models by Sumpter [25]. While thermodynamically correct, these continuum models are challenging to implement in homogeneous tissues, and become even more so in the spatially heterogeneous tissues relevant to biology. They also lack the cell-level detail to elucidate the impacts of ice propagation rates and damage on key cell types and tissue substructures. Therefore, while continuum models of ice propagation give a good overview of the rates at which ice may form throughout a tissue, they lack the precision and detail needed to understand why some parts of tissues survive and others do not under any particular freezing protocol.

Agent-based models have been used in computer science and social science to study self-organizing computer programs, robots and individuals [33–36]. They have been applied in biological systems to model capsule-shaped bacteria [37], molecular systems in biology [38], and cancer and tumour growth and mechanics [39,40]. Agent-based models are generally characterized by self-determining objects called agents equipped with protocols for inter-agent interaction, such as the ability to recognize and distinguish the features of other agents [41].

Our long-term objective is to implement a histologically accurate model of tissues undergoing cryopreservation, enabling one-to-one mapping of experimental and model results, and promoting deeper understanding of the effects of tissue structure on cryo-survival. In this paper, we couple the cell-based multicellular stochastic model introduced in [23] with a modern agent-based method to model lattice-free ice propagation in three-dimensional lattice-free tissues and account for the solidification of water into ice in cells. In our approach, each cell in the tissue is considered as an agent using the open source software PhysiCell [40], a multicellular system simulator which is designed to model tissues involving many interacting cells in multi-substrate three-dimensional microenvironments. In addition to expanding existing models from two-dimensional lattices to three-dimensional lattice-free settings, our implementation of PhysiCell includes a novel model of the important mechanical effects of ice formation and expansion in a relatively large tissue, a well-understood mechanism of cryoinjury in tissues [42].

## 2. Methods

### 2.1. Models

#### 2.1.1. Overview

Our model aims to capture both cell- and tissue-level details of a tissue undergoing IIF. Briefly, at the individual cell level (i.e. for each agent), we capture time-dependent cell characteristics including the frozen/unfrozen state, position, volume, intracellular concentration, intracellular viscosity (in the unfrozen state) or final volume in the frozen state. At the tissue level, cell states, volumes, lattice-free locations and number of neighbours inform mechanical deformation models that then reshape the tissue or infer tissue damage when intercellular connections are disturbed. Here, we present and validate an implementation of an ice formation and intercellular propagation model based primarily on the work of Irimia & Karlsson [22,23]. This ice formation and propagation model requires estimates of the intracellular viscosity as a function of time, which in turn is determined by the intracellular solute concentration at a given temperature. Thus, next, we present and validate a standard model of transmembrane water transport that will also primarily define the cell volume as a function of time, and supplement this model with the water-to-ice volume shift as a function of solute concentration. Next, we present parameter identification for a non-dimensional time variable, *τ*. Finally, we show how this cell volume function is then coupled with an intercellular mechanics model. Note that we do not model extracellular ice formation because during cryopreservation of tissues extracellular ice is formed, the excluded solutes depress the freezing point in the remaining medium [43], and as such the surrounding medium around cells and tissues is generally liquid until the eutectic or glass transition temperature [44].

#### 2.1.2. Stochastic model of intracellular ice formation and propagation

IIF is a stochastic event [45]. To capture the behaviour that ice preferentially propagates from a cell with ice to a cell without ice, Irimia & Karlsson [22,23] developed a model where the mechanisms of IIF were described by two independent stochastic processes: *J*^i^, a rate describing the class of all mechanisms that cause IIF independent of their neighbouring cells’ states, and *J*^p^, the total rate of intracellular ice propagation across the interface between a frozen and an unfrozen cell. Hence, the total rate of IIF in unfrozen cell *j* is the sum of the rates associated with these two independent stochastic functions. In this present paper, we assume that if cell *j* has *k*_*j*_ frozen cell neighbours the intracellular propagation rates are not cell or location dependent. Then, we have

**Figure 1.**
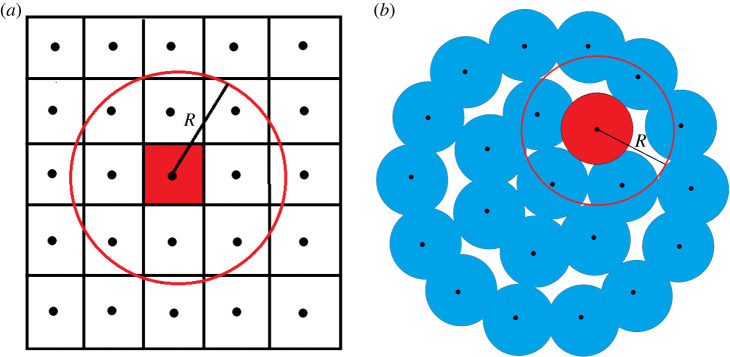
Two-dimensional representation of neighbourhood geometry. Cells which have a centre within a ball of radius *R* and the same centre of the cell of interest *j*, here indicated in red, are considered to be the neighbour cells. (*a*) A ‘square’ cell configuration where the number of neighbours for a central cell *j*, *k*_*j*_ = 8. (*b*) A two-dimensional ‘single layer’ configuration of round cells, the number of neighbours for the red cell *j* is *k*_*j*_ = 4. We account for a spherical cell morphology by allowing for up to 20% cell radius overlap of our spherical cell agents.

2.1 $$J_{j}=J_{j}^{i}(t)+k_{j}J_{j}^{p}(t),$$

where *J*_*j*_ represents all possible observations of IIF per unit time per cell. We define the *j*th cell’s neighbourhood as the cells which have a centre within a ball of some radius *R* around the centre of the *j*th cell, as indicated in figure 1. This neighbourhood definition is inspired by the fact that independent ice formation may be due to surface catalysed nucleation mediated by intracellular ice in an adjoining cell. The probability that an unfrozen cell *j* freezes in a time interval Δ*t* can be described by the Poisson process

2.2 $$p_{\;j}^{\mathrm{IIF}}=1-\exp\left[-\int_{t}^{t+\Delta t}J_{j}^{i}(t)+k_{j}J_{j}^{p}(t)\;dt\right].$$

To non-dimensionalize the above equation and simplify the computational model, a non-dimensional time *τ* can be introduced [22]:

2.3 $$\tau =\int_{0}^{t}J^{i}\;dt.\;$$

Also, a non-dimensional ice propagation rate can be defined as

2.4 $$\alpha =\frac{J^{p}}{J^{i}}.$$

In practice, the larger *α* is, the faster the ice propagates from frozen neighbour to unfrozen neighbour, and is discussed in more details in [22,23].

#### 2.1.3. Monte Carlo model

In small systems, the ice propagation model can be solved explicitly using a Markov chain (for one-dimensional systems, see [22,23]; for an example of a two-dimensional system, see appendix A). But this solution does not scale well, with numbers of states approximated by 2^number of cells^. As an alternative to the Markov chain approach, Monte Carlo methods are used to approximate the probabilities of outcomes based on repeated random sampling. Our development of the Monte Carlo method closely follows Irimia & Karlsson [22,23]. Briefly, the probability that an unfrozen cell *j* ∈ {1, …, *N*} may freeze within a time interval Δ*t* is defined by the sum of the probabilities of spontaneous and propagation mechanisms. Following Irimia & Karlsson [23], using equations (2.2)–(2.4), we have

2.5 $$p_{j}^{\mathrm{IIF}}(\Delta \tau )\approx p_{j}^{i}+p_{j}^{p}\approx (1+k_{j}\alpha )\Delta \tau ,$$

where a sufficiently small Δ*τ* must be chosen to ensure at most one change of state (IIF event) per time step. Irimia and Karlsson suggest

2.6 $$\Delta \tau =\frac{\varepsilon }{(1+n\alpha )N},$$

where *n* is the maximum number of nearest neighbours, and ε is a small probability bound for the IIF probability in the tissue during time-interval Δ*τ* [23].

To solve this, we implemented the Gillespie algorithm [46], adopting the approach by Irimia & Karlsson [23] and Stott & Karlsson [47] (see appendix B for details). To validate our implementation, we analytically solved the associated Markov model to determine the probability of each of the six states as a function of time for a 2 × 2 cell structure (see appendix B, figure 8), and we compared exact Markov and numerical Monte Carlo models. Using parameters from Irimia and Karlsson (e.g. *α* = 10.4), we compared mean results from 10^5^ Monte Carlo iterations with 1744 total time steps.

#### 2.1.4. Water transport model

The rate of IIF, *J*^i^, is a function of intracellular viscosity and the melting point of the cytosol (cf. equations (2.10) and (2.11), below). Our model also attempts to capture the intercellular forces within a tissue during cooling. At moderate cooling rates and higher, these phenomena are governed by the water content and state of the cell, which decreases in response to the increasing extracellular osmolality [48]. Here, we employ the non-equilibrium water transport model introduced by Mazur [12]. Transport across the cell membrane occurs due to differences in chemical potential between the intracellular and extracellular solutions [49]. Hence, the kinetics of water loss at sub-zero temperatures is

2.7 $$\frac{dV}{dT}=-\frac{L_{p}ART}{Bv_{w}}\left[\ln\left(\frac{V-V_{b}}{\left(V-V_{b})+v_{w}(v_{s}n_{s}\right)}\right)-\frac{\Delta H_{f}}{\bar{R}}\left(\frac{1}{T_{R}}-\frac{1}{T}\right)\right],$$

where *V* is cell volume; $\bar{R}$, the universal gas constant; *T*, absolute temperature; *A*, surface area of the cell; *B*, the cooling rate; *v*_w_, partial molar volume of water; *V*_b_, osmotically inactive cell volume; *v*_s_ = 2, the dissociation constant for NaCl; *n*_s_, number of moles of salt in the cell; Δ*H*_*f*_, the latent heat of fusion of water; *T*_*R*_, the equilibrium freezing temperature for pure water (273.15 K); and *L*_p_, the hydraulic conductivity. The temperature dependence of *L*_p_ is given by an Arrhenius model with

2.8 $$L_{p}(T)=L_{p_{g}}\exp\left(-\frac{E_{L_{p}}}{R}\left(\frac{1}{T}-\frac{1}{T_{R}}\right)\right),$$

where *L*_p__g_ is the hydraulic conductivity of water at *T*_*R*_ and *E*_L__p_ is the activation energy for the water transport process. For commonly implemented constant-rate cooling protocols, the temperature as a function of time *t* is given by

2.9 $$T(t)=T_{0}-Bt\;\mathrm{and}\;T_{0}=T(0),$$

where the transformation from temperature to time can be implemented using the cooling rate *B*.

#### 2.1.5. Non-dimensional time

Up to now, our model was defined in terms of the time-like variable *τ*, but in order to compare the introduced model with experimental results, we need to obtain *τ* as a function of time or temperature. From equation (2.3), *τ* can be evaluated if the cell surface area *A* and the independent rate *J*^i^(*T*) are known. Karlsson *et al.* [16,19,20,28] define

2.10 $$J^{i}(T)=A\Omega_{0}\frac{\eta_{0}}{\eta }{\left(\frac{T}{T_{f_{0}}}\right)}^{\frac{1}{2}}\exp\left[-\frac{\kappa_{0}}{T^{3}{\left(T_{m}-T\right)}^{2}}{\left(\frac{T_{f}}{T_{f_{0}}}\right)}^{4}\right],$$

where the subscript ‘0’ refers to isotonic conditions, Ω_0_, *κ*_0_ are kinetic and thermodynamic coefficients, respectively,

2.11 $$\eta \;:=\eta_{w}\exp\left(\frac{2.5\phi_{s}}{1-\varphi \phi_{s}}\right)\;$$

is the cytoplasmic viscosity as a function of the volume fraction of salt, *ϕ*_s_, φ = 0.609 is the hydrodynamic interaction constant [50], and

2.12 $$\eta_{w}=0.139{\left(\frac{T}{225}-1\right)}^{-1.64}$$

is the temperature-dependent viscosity of pure water [19].

The equilibrium freezing temperature of the cell cytoplasm [18]

2.13 $$T_{f}={\left[\frac{1}{T_{f_{0}}}-\frac{R}{\Delta H_{f}}\ln(x_{w})\right]}^{-1}$$

is a function of the mole fraction of water,

2.14 $$x_{w}\;:=\frac{n_{w}}{n_{w}+2n_{s}},$$

and where *n*_w_ and *n*_s_ are moles of intracellular water and dissociated salt, respectively. Note that it is this mole fraction that requires the water content of the cell, modelled above using equations (2.7)–(2.9), and hence the salt volume fraction is updated when the cell water content changed.

#### 2.1.6. Ice expansion and tissue remodelling

As a first approximation for capturing the potential disruption of tissues due to the sequential solidification of the intracellular spaces, we used the following approach. First, we modelled the propagation of ice in the tissue structure as described above. Then for any particular Monte Carlo simulation, we then made a list of times *τ*_*j*_ at which cell *j* solidified. Since PhysiCell provides the property for the users to define and modify some of the existing function and libraries, we created rat hepatocyte custom data, cell type and cell rules functions. We assigned a true or false frozen state to each cell as its property. Next, for each cell, our code checks if the cell is frozen or not, and for a non-frozen cell we used model equation (2.7) evaluated at the PhysiCell current global simulation time to determine the intracellular water content at this time and we updated all the variables that are water content dependant. At this time, equation (2.3) was evaluated to obtain the dimensionless time associated with this cell. We compared this dimensionless time to the related value in the list of dimensionless times obtained from the Monte Carlo method. If these two dimensionless times are very close then the cell is considered to be solidified at this PhysiCell current time. The PhysiCell global simulation time is updated based on diffusion time steps. If the cell solidified in this time step, then the remaining intracellular water was assumed to turn entirely into ice with a volume expansion of 1.09. We assumed that unfrozen cells remain attached to frozen cells until they become frozen, or until they are separated by a distance described below.

Running in real (dimensional) time, we used the PhysiCell mechanical force model with default parameters for our tissue construct [40], except where the volume state of the *j*th cell was changed at time *t*_*j*_ to have its new, expanded volume. This process was repeated for all *j* ∈ {1, …, *N*}, and the force model was allowed to run as usual throughout the entire freezing experiment.

PhysiCell uses a simple potential function to avoid cell lattice effects. It updates the velocity and eventually cell’s position according to force-based cell–cell interaction mechanics. Using this built-in model with the cell–cell adhesion strength is considered to be 30 pN, repulsion strength is 750 pN and the maximum adhesion distance is 1.25 μm. If the cell membranes do not touch then there is no interaction between cells. However, if the distance between two cell centres is less than the sum of their radii, then two cells may overlap. Note that for simplicity of presentation (i.e. it is difficult to visualize the mechanical disruption in three-dimensional tissue constructs), we only performed this procedure on our 22-cell hepatocyte monolayer discs.

### 2.2. Implementation in code

The original core library files for PhysiCell were used with no modifications. We modified the custom configurations file by setting Δ*t*_mechanics_ = 0.001 s and Δ*t*_diffusion_ = 0.001 s. The time-step size or phenotype processes was the default value. Dirichlet boundary conditions were used on the cuboid geometry of the computational domain. Global PhysiCell time was updated and saved based on diffusion time steps. However, no reaction–diffusion modelling took place in this work. PhysiCell custom modules were replaced by our custom code which include functions such as volume, geometry, water transport, neighbourhood, Monte Carlo algorithms and dimensionless time functions. We developed Matlab and Mathematica codes for post-possessing and visualizations. The numerical integration is implemented using composite trapezoidal rule. All custom code is included in the GitHub repository [51].

### 2.3. Numerical experiments

In order to test and validate the introduced model, we perform some numerical experiments in the following sections. Therefore, we implement the model to study ice propagation in rat hepatocyte tissue constructs. The biophysical parameters for hepatocytes are given in table 1 and were taken from [19,52]. First, we start by analysing the relationship between non-dimensional time, dimensional time and temperature in one cell rat hepatocyte in §3.1. Then, we study the effect of the magnitude *α* and the maximum number of neighbours for each cell on the Monte Carlo model in predicting the probably of ice formation in a disc monolayer tissue in §3.2.1. To study the ice propagation path, we consider a pre-frozen cell located as indicated in figure 4, in this section. Two cases were studied: (i) a pre-frozen cell in the centre and (ii) a pre-frozen cell in the outside boundary of the monolayer. For each case, the Gillespie method was used with 100 iterations and the neighbour list for each cell is defined based on cell to cell membrane contacts. In order to study the mechanical disruption of volume expansion, we performed a small set of numerical experiments on a two-dimensional tissue structure to enable better visualization. In these experiments, the sudden expansion of intracellular water into ice was modelled using PhysiCell’s built-in mechanics models experimentally validated for liver structures [53].

**Table 1.** Table of parameters, descriptions and values.

parameter	description	value
*J*_*j*_	rate of formation of ice in cell *j*	variable
*R*	radius of neighbourhood of cell	variable
$J_{j}^{i}$, $J_{j}^{p}$	rate of formation of ice in cell *j* due to intracellular and extracellular processes, respectively	variable
*t*	time	variable
$p_{j}^{\mathrm{IIF}}$	probability that ice has formed in cell *j*	variable
*k*_*j*_	the number of frozen neighbours of cell *j*	variable
*τ*	non-dimensional time	variable
*A*	cell surface area	1.412 × 10^3^ μm^3^
*α*	non-dimensional ice-propagation rate	variable
*N*	number of cells	variable
*M*	number of possible states	*M* = 2^*N*^
*X*	system state	*X* ∈ (1, …, *M*)
*P*	state probability vector	variable, *P* = {*p*_*i*_}_*i*∈*X*_
*Q*	state transition matrix for the Markov process	variable, $Q\in R^{M}\times R^{M}$
*f*_*j*_(*τ*)	state of cell *j* at time *τ*	variable
*L*_pg_	cell membrane hydraulic conductivity at reference temperature *T_R_*	1.5 × 10^−12^ m^3^ N^−1^ s^−1^
*E*_Lp_	activation energy of the hydraulic conductivity	3.42 × 10^2^ kJ mol^−1^
*T*_*R*_	reference temperature	273.15 K
$T_{\;f_{0}}$	freezing point of cytoplasm at isotonic conditions	272.63 K
*T*_0_	the initial temperature at time zero	272.15 K
*v*_w_	molar volume of water	1.8 ×10^13^ μm^3^ mol^−1^
*v*_s_	dissociation constant for NaCl	2 mol
Δ*H*_*f*_	latent heat of fusion of water	5.94124 × 10^16^ μm^3^ atm mol^−1^
*v*_b_	osmotically inactive volume fraction	0.51 (unitless)
*V*_*b*_	osmotically inactive volume	$v_{b}\times V_{\mathrm{iso}}$
$\bar{R}$	gas constant	8.314 J mol^−1^ K^−1^
*B*	cooling rate	variable
Ω_0_	kinetic ice formation parameter	1.1 × 10^10^ m^−2^ s^−1^
*κ*_0_	thermodynamic ice formation parameter	1.4 × 10^9^ K^5^ s
*η*	cytoplasmic viscosity	variable
*η*_*w*_	viscosity of pure water	variable
*T*_*f*_	equilibrium freezing temperature of cytoplasm	variable
*x*_*w*_	mole fraction of intracellular water	variable
*n*_*w*_, *n*_*s*_	moles of intracellular water and salt, respectively	variable

Rat hepatocyte spheroids are generally 100–200 μm in diameter, are roughly spherical, and contain approximately 100 hepatocytes [54]. In §3.2.2, we emulated this in our model by creating a three-dimensional lattice-free rat hepatocyte spheroid of approximately 100 μm in diameter with 103 cells and started our simulations with a pre-frozen cell on the border of the spheroid.

Next in §3.3, we consider a larger three-dimensional homogeneous tissue slab comprised of rat hepatocytes with a lattice-based cell arrangement of 31×11×7 cells (approx. 651 μm × 231 μm × 147 μm) with one initial frozen cell seeded in a corner (figure 7*a*′). The Gillespie method was applied for 100 iterations. In this construction, the maximum number of neighbours for each cell is *k* = 8, which becomes reduced if the cell is on a face, edge or corner of the tissue slab.

The Gillespie method only updates the simulation when an event occurs, rather than at every time step. While the Monte Carlo method may require a large number of iterations to converge to an accurate solution, especially for high-dimensional or complex systems. The efficiency of the Gillespie algorithm lies in its ability to focus computational effort only on relevant events, rather than uniformly throughout the simulation. This targeted approach reduces the number of iterations needed to accurately simulate the system’s behaviour, particularly in scenarios where events occur infrequently or with varying rates.

## 3. Results and discussion

### 3.1. Non-dimensional time validation in single cells

**Figure 2.**
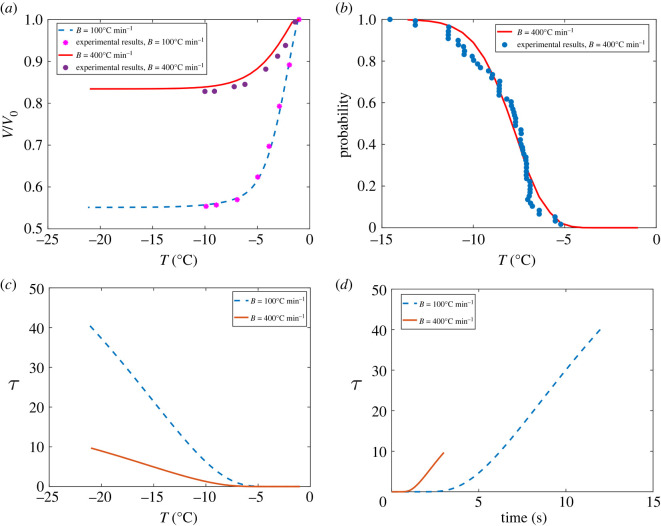
Comparison of theoretical and experimental results for individual rat hepatocytes, and the resulting *τ* versus time and *τ* versus temperature relationships. The experimental results were digitized from [19].

In figure 2, the normalized cell volume is plotted as a function of temperature for an average rat hepatocyte cell. As can be seen from figure 2, the cell water content depends strongly on the cooling rate. At a moderate cooling rate such as *B* = 100°C min^−1^, the cell loses almost all of its water, while for a rapid cooling rate, *B* = 400°C min^−1^, the cell retains about 85% of its original water content. Figure 2 illustrates the monotonic (and therefore invertible) relationship between *τ*, time and temperature for rat hepatocytes. Here, the required parameters for updating the *J*^i^ model (equation (2.10)), specifically the water volume of the cell that manifests in equation (2.13), are updated from the water transport model (equation (2.7)). To compare or match model predictions of kinetics, given in terms of the non-dimensional time variable *τ*, with experimental data that describe probabilities of ice as a function of temperature, we used the relationship shown in figure 2 to convert between temperature units and non-dimensional time.

Figure 2 shows the comparison between theoretical and previously reported experimental results obtained from using a cryomicroscopy system at different cooling rates [52]. The theoretical IIF probability results first were obtained based on dimensionless *τ*, then *τ* was converted to temperature using figure 2. The small difference between our results and the experimental results indicates a good approximation using our method. The probability of IIF was 0.0% at cooling rates slower than 80°C min^−1^ and that the cumulative probability of IIF does not converge to 100% until the rate of cooling exceeds 130°C min^−1^. This indicates that our results are consistent with corresponding results shown in [19]. However, as reported in [52], the maximum cumulative incidence of IIF is a function of seeding temperature, *T*_0_, and the lower seeding temperature results in higher maximum cumulative probability.

### 3.2. Rat hepatocyte disc monolayers and spheroids

#### 3.2.1. Disc monolayers

**Figure 3.**
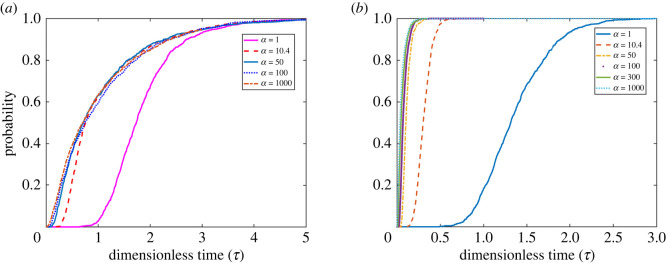
The probability of the state that all the 22 cells are frozen in disc monolayer. Probability of IIF for 22-cell hepatocyte monolayer with (*a*) *R* = 21 μm and (*b*) *R* = 22 μm. Note that the hepatocyte diameter used is 21 μm and up to 20% cell radius is allowed to overlap.

In this example, we first consider a lattice-free disc-shaped monolayer of rat hepatocytes with 22 cells, figure 1*b*. Simulations were conducted to study the impact of non-dimensional ice propagation parameter *α*, as well as *R*, the radius of the cell neighbourhood, or *k*, the maximum number of neighbours, on the model. The cell properties are given in table 1. In figure 3, two different values of *R* = 21 or *R* = 22 μm with associated *k* = 2 or *k* = 7, respectively, are studied. The probability of the state that all the cells are frozen is given by $p_{22}=1-\sum_{i=0}^{21}p_{i}$, and is indicated in figure 3. These results indicate that when *R* and *α* decrease, the intracellular ice propagation, *J*^p^, decreases as well. That means for the two different *R* and the same *α*, the number of cells that freeze from ice propagative IIF is higher for higher *k*. Thus, *J*^p^ depends on the number of neighbour cells, *k* and *α*.

As can be seen from figure 3, a smaller *τ* is obtained for higher *R*. This demonstrates that the higher number of connections between cells induces more ice formation due to ice prorogation and reduces the ice formation due to the intracellular ice nucleation, *J*^i^. For fixed *R* and different *α*, there is a threshold such that increasing *α* does not effect the solution, and figure 3 illustrates that the threshold value for both cases is approximately 50. This is due to the higher number of intercellular connections and is associated with the increased number of gap junctions that facilitate the ice propagation [21]. However, *J*^i^ is independent of the number of neighbour cells *k* and *α*. Acker and McGann proposed that intracellular ice propagation is harmless and the damage is only caused by intercellular ice nucleation, *J*^i^ [55,56]. This suggests that for high cooling rates the IIF damage is reduced by minimizing *J*^i^.

**Figure 4.**
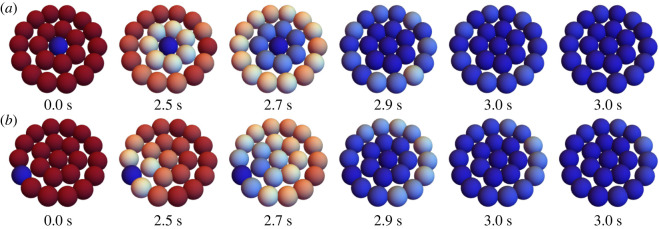
Rat hepatocyte monolayer disc ice probability. The monolayer cooled at 100°C min^−1^ with an initial cell frozen (indicated by blue) in the centre (*a*) or the corner (*b*) after 100 numerical trials. Unfrozen cells are indicated with red, and the probability is indicated by the colour with more blue indicating a higher probability of ice in that cell at that timepoint. For simplicity, cell volumes and mechanic disruptions are not shown here.

The probability that the ice propagates in a certain path in the monolayers is indicated in figure 4 for a pre-frozen cell in the centre and at the outer layer of the tissue. This figure indicates the probability distribution path in the tissue for both cases. We found that when the pre-frozen cell is in the centre, the ice propagates symmetrically towards outside of the monolayer. When the pre-frozen cell was on the border, the ice is more likely to propagate to the centre of the monolayer. These results align with the intuition that ice propagates through highly networked cells, and thus does not propagate quickly along the outer surface of the tissue structure, and may have implications for specific tissue geometries where cell networking changes based on cell line or tissue type, such as those with vasculature or other substructures.

**Figure 5.**
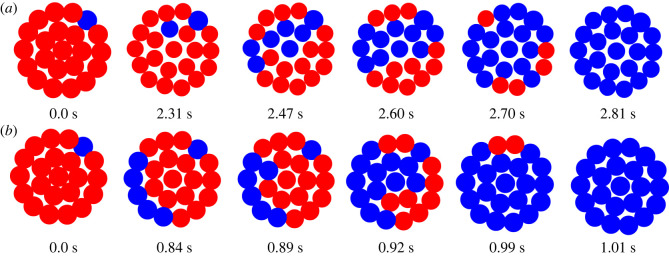
Rat hepatocyte monolayer disc mechanics. The results for single numerical trials with an initial cell frozen in the corner, indicated by blue, and unfrozen cells indicated by red, cooled at 100°C min^−1^ (*a*) or 400°C min^−1^ (*b*). Cells lose more water over time at slower cooling rates, and do not lose water upon freezing.

Figure 5 shows the volume change during dehydration induced by both moderate and fast cooling rates. Upon the solidification, two things change. First, the cell ceases to lose more volume, and second, any intracellular water is converted to ice at the expansion of 1.09. In our results, it is clear that there are two different ice propagation mechanisms at work at the moderate and fast cooing rates. For *B* = 100°C min^−1^, ice propagates sequentially from cell to cell, creating a region of non-reactive cells that do not continue to shrink. This causes potential fissure points across the plane of ice propagation. By contrast, at the fast cooling rate (*B* = 400°C min^−1^), there is less order in the position of cells that solidify. There is also reduced volume loss due to the shorter time course. This manifests in more individual cells separating from their neighbours, suggesting that at higher cooling rates, a different mechanism of tissue level ice damage may occur. Note, however, as we observe here and below, ice propagates preferentially through well-networked cells. This suggests that there may be cases where some tissue structures, or even some specific cell types, that are likely to get ice and cause mechanical disruption to that portion of the tissue. Moreover, there may be more realistic tissue structures that are more sensitive to these disruptions. Finally, our approach is a first approximation of the actual mechanical dynamics of solidification in tissues. One expects, for example, temperature and state dependence of intercellular force mechanics, as well as the dependence on the state and domain of extracellular ice. If some of the extracellular space is already solid, cells may be bound in complicated ways to each other and already solidified portions. More research and careful experimentation is needed on this aspect.

#### 3.2.2. Hepatocyte spheroids

**Figure 6.**
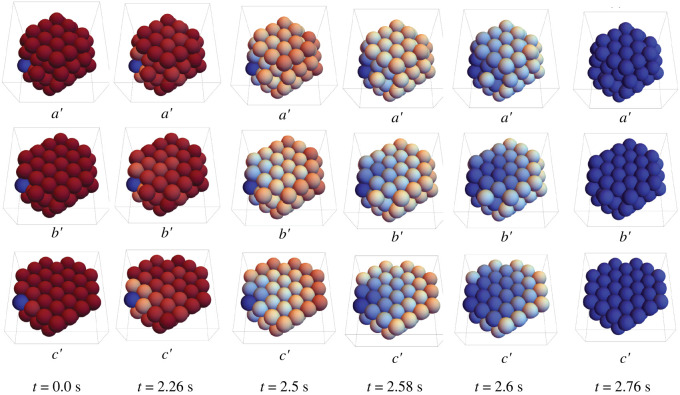
Probability distribution of ice propagation in different time steps of rat hepatocyte spheroids with 103 cells and a pre-frozen (blue) cell in the corner of the spheroid with *α* = 10.4 and *B* = 100°C min^−1^. Red indicates no likelihood of ice, blue indicates a 100% likelihood of ice. The whole spheroid is indicated in *a*′, one layer has been removed in *b*′, and two layers have been removed in *c*′.

The probability of ice propagation in three different layers of the spheroid is illustrated in figure 6 at different time steps. In figure 6, *a*′ indicates the whole spheroid with 103 cells, while in *b*′ and *c*′ one and two layer from the top of the spheroid have been removed, respectively, in order to present more views of the inside the spheroid. In this case, analogous to the monolayer case, ice propagates towards the centre of the spheroid where there are more connections between cells.

### 3.3. Hepatocyte tissue slab

**Figure 7.**
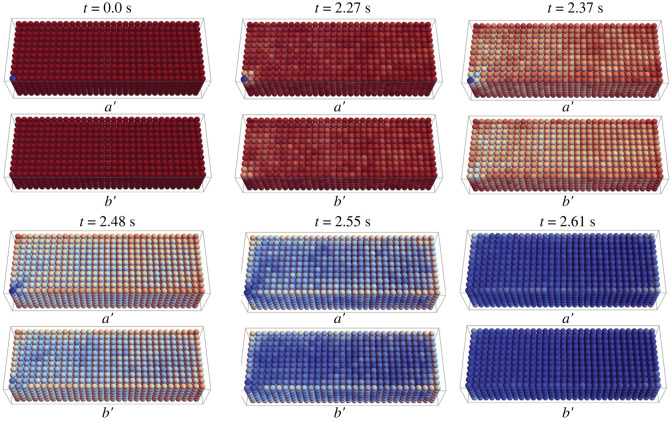
Probability distribution of ice propagation in different time steps of a large tissue with 2387 cells and a pre-frozen cell in the corner of the tissue with *α* = 10.4 and *B* = 100°C min^−1^. The whole tissue is indicated in *a*′, and one layer has been removed in *b*′.

Figure 7 indicates the probability of ice as a function of location in the tissue. Our results confirm that the ice propagates most readily in the tissue centre, where the average number of cells connected is higher. In particular, the most rapid propagation in the slab happens with the centre-seeded cell position, with the face-seeded cell position coming in second, and the corner-seeded cell position being the slowest. Moreover, the ice propagation in the face- and corner-seeded slabs typically progresses towards the centre of the tissue and then outward as opposed to along an edge or face. This phenomenon reflects the relative connectivity of cells at the centre. In more realistic tissues, the intercellular connection numbers may vary spatially, and our current model is uniquely capable to address this. Note, also, that as the tissue size increases, the average connectivity between cells increases, and depending on *α* the rate of complete ice formation in a well-connected large tissue could be higher than that of a less-well-connected small tissue.

**Figure 8.**
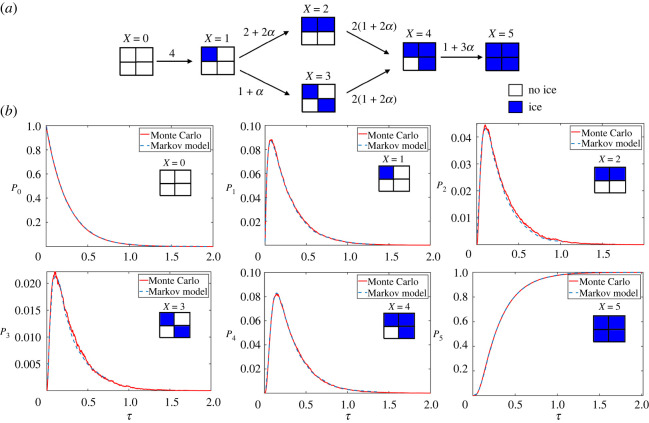
(*a*) All possible states for a 2 × 2 cell construct with *X* = 0, …, 5 indicated above each state. Each state depends on the previous state. Symmetries are not shown, but are indicated by the non-dimensional rates given above the arrows. For example, there are four symmetric singlet states, so the rate to go from state *X* = 0 to state *X* = 1 is 4. There is only one way to go from a singlet state (*X* = 1) to the opposite corner singlet state (*X* = 3): either via the independent mechanism at non-dimensionalized rate 1, or via the propagative mechanism at rate *α*. By contrast, there are two symmetrically equivalent ways to arrive at state 2, each with an independent rate of 1 and a propagative rate *α*, totalling 2 + 2*α*, etc. (*b*) Exact (Markov chain) and numerical (Monte Carlo) predictions of the probabilities of each state in the 2 × 2 structure, with propagation rate *α* = 10.4, implemented within the PhysiCell agent-based code. Inset is the associated diagram for each state, with blue indicating ice.

Precision cut liver slices (PCLSs) have significant clinical and experimental relevance, but currently do not have an acceptable cryopreservation protocol [57]. PCLSs are typically 2–5 mm diameter cylinders of 250 μm thickness [58]. Therefore, the current slab model captures between 5% and 1% of the total volume of a 2–5 mm diameter PCLS, respectively. Both PhysiCell and our Gillespie Monte Carlo method scale linearly with cell number and both take advantage of OpenMP parallelization. In fact, PhysiCell has been implemented in very high-performance computing centres, suggesting that scale up to full size PCLSs is well within computational reach, even with the requirement of multiple numerical experiments to inform the Monte Carlo model [59].

More anatomically realistic implementations of liver tissue using cell-agents can be implemented. For an example, recently Wang *et al.* [53] implemented realistic hepatic lobules using PhysiCell and even accounted for sources and sinks of transport via the vascular system. Their purpose was tumour modelling, but adaptation to using our model is relatively straightforward. In fact, there is much interest in modelling ice propagation in tissues and organs for the purposes of tumour ablation, known as cryosurgery [60]. Ice propagation in complex tissues is thought to occur first in the vasculature, followed by nucleation in capillaries where it then spreads throughout the tissue or organ [61]. Therefore, the model described by Wang *et al.* is ideally situated to be adapted using our current methods to ice propagation modelling, both from the preservation side and from the tumour ablation side.

In large tissues, different cooling rates are experienced in different parts of the tissue. In the current paper, it was assumed that there were no thermal gradients in the tissues, a reasonable assumption for tissues of diameter of less than 150 μm at the moderate cooling rates studied here. However, the sensitivity of the probability of IIF on the temperature of the cell, shown in figure 2, suggests that in larger tissues such as PCLSs or organs accounting for the heat transfer in the system will become important. Moreover, in the current paper, the extracellular medium for each cell was assumed to be in a thermodynamic equilibrium that is solely a function of temperature. Anderson, Benson and Kearsley show that there can be a spatial dependence of concentration in the extracellular milieux during cooling [62,63] on the individual cell level, and Warner *et al.* suggest that, even at superzero temperatures, there is a complicated interaction between solute, solvent and cellular transport as the size of the tissue increases [64]. Therefore, future work in larger tissues should pay close attention to the possible distribution of both heat and concentration, their effects on the intracellular state of each agent, and in turn, the likelihood that any cell may experience IIF. Wang *et al.* used the finite volume-based reaction–diffusion equation solver associated with PhysiCell to describe the transport of sugars and oxygen for cell support, but this mechanism can be repurposed to capture heat and solute transport relevant to cryobiological applications.

## 4. Conclusion

This study applied a lattice-free agent-based model in combination with a stochastic model for ice formation and propagation in an arbitrary tissue. This method was implemented with the advanced and robust open source package PhysiCell. In agent-based models, cells, as agents, can move and interact with each other freely, and in our implementation, the movement was influenced by the expansion of intracellular water as ice. The probability of ice formation in each cell at a given time is obtained based on two non-dimensional parameters *τ* and *α*. The non-dimensional parameter *τ* in this model makes it possible to calculate the probability independent of cell type. However, to obtain the real time instead of *τ* which is dependent on the cell type, the information about the mechanism of ice formation inside the cell is needed. Here, we proposed a formulation to compute *τ* in the absence of CPA. We showed that *τ* is a function of cell type and cooling rate. We conclude that there is a threshold value of *α* = 50 for rat hepatocytes, and that increasing the value of *α* beyond this threshold does not affect the probability of IIF. However, the actual value of *α* depends on the intercellular connections in different tissue types. The higher connection between cells facilitates the ice formation due to ice propagative *J*^p^ and reduces the ice formation due to intracellular ice nucleation *J*^i^. This study has also shown that ice is most likely to propagate into the centre of the tissue where there are a higher number of neighbour cells and connections. The proposed model approximation also shows potential for other problems which will be examined in the future, such as ice expansion and propagation in liver tissue with different cell types, mass and heat transport and the presence of cryoprotective agents.

## Data Availability

The data are provided in the GitHub repository [51].

## Appendix A. Ice propagation as a Markov process

For illustration, here we extend the results from Irimia & Karlsson [23] and consider a 2 × 2 cell structure. The possible states and their related rates are illustrated in figure 8, accounting for symmetries. In this case, for each cell *j*, the maximum number of nearest neighbours is three, including the diagonal neighbour. To determine the probabilities of each state as a function of time in this geometry, an analytical method such as a Markov chain model is used. Assume that the state *X* ∈ {0, …, *M*} is associated with probability *p*_*X*_(*t*), where *M* is the maximum number of possible states of observation at a given time. The IIF state *X* includes the frozen and unfrozen individual cells in identical tissue constructs. Since the state space {0, …, *M*} is finite, the transition probability distribution can be indicated by a transition matrix. In the case of *M* = 5, this becomes

A1 $$Q(\alpha )=\left(\begin{array}{ccccc} -4 & 0 & 0 & 0 & 0\\ 4 & -3(1+\alpha ) & 0 & 0 & 0\\ 0 & 2(1+\alpha ) & -2(1+2\alpha ) & 0 & 0\\ 0 & \left(1+\alpha \right) & 0 & -2(1+2\alpha ) & 0\\ 0 & 0 & 2(1+2\alpha ) & 2(1+2\alpha ) & -(1+3\alpha ) \end{array}\right).$$

Therefore, if *P* = {*p*_*X*_}_*X*=0,…, *M*−1_ presents a vector of all state probabilities except *p*_*M*_ which is $p_{M}=1-\sum_{i=0}^{M-1}p_{i}$, then the probability of each state can be obtained by solving a Kolmogorov differential equation:

A2 $$\frac{dP}{d\tau }=Q(\alpha )P,\;P(0)={\left(1,0,\ldots ,0\right)}^{T}.$$

However, due to computational complexity, the size of system (A 2) increases exponentially with tissue size and quickly becomes intractable, so a different approach must be used.

## Appendix B. Solution of the Monte Carlo problem

We define the ice-state of cell *j* by

B1 $$f_{j}(\tau )=\left\{ \begin{array}{cc} 0 & \text{if cell}\;j\;\text{is unfrozen at time}\;\tau ,\\ 1 & \text{if cell}\;j\;\text{is frozen at time}\;\tau ,\; \end{array}\right.$$

where *f*_*j*_ is a property of the *j*th cell-agent. For every unfrozen cell at each time step Δ*τ*, a random number *r*_*j*_ is generated from the uniform distribution on the interval (0, 1). If $r<P_{j}^{\mathrm{IIF}}$ the cell *j* considered as frozen. However, accurate results require a high number of iterations or experiments. This makes the Monte Carlo method computationally expensive.

To overcome this problem, Karlsson & Irimia [23] propose using the Gillespie algorithm [46,47], which estimates both the reaction (conversion to IIF) location and the time of reaction by two random variables *r*_1_, *r*_2_ compared with the uniform distribution on (0, 1). This means that for a simulation of *N* cells, only *N* time steps are needed, a considerable saving in computational time.

To implement this algorithm, assume that the overall rate at time step *i* is as follows:

B2 $$a_{0}=\sum_{\;j=1}^{N}(1-f_{j})(1+k_{j}\alpha ),$$

where *N* is the number of cells in the tissue. Then, the next time step is

B3 $$d\tau =\frac{1}{a_{0}}\ln\left(\frac{1}{r_{1}}\right),$$

and the specific cell is to be frozen at the time *τ* + d*τ* is given by the *j* that satisfies

B4 $$\sum_{m=1}^{\;j-1}(1-f_{m})(1+k_{m}\alpha )<a_{0}r_{2}\leq \sum_{m=1}^{\;j}(1-f_{m})(1+k_{m}\alpha ).$$

We implemented both the standard Monte Carlo and Gillespie algorithms within the construct of PhysiCell, and note that when *N* < 100, their computational cost is similar.
